# Optical Multistability in the Metal Nanoparticle–Graphene Nanodisk–Quantum Dot Hybrid Systems

**DOI:** 10.3390/nano10091687

**Published:** 2020-08-27

**Authors:** Mariam M. Tohari, Moteb M. Alqahtani, Andreas Lyras

**Affiliations:** 1 Department of Physics, College of Science, King Khalid University, Abha 61413, Saudi Arabia; moalqhtani@kku.edu.sa; 2 Department of Physics and Astronomy, College of Science, King Saud University, Riyadh 11451, Saudi Arabia; alyras@ksu.edu.sa

**Keywords:** optical multistability threshold, unidirectional ring cavity, giant self-Kerr nonlinearity, metal nanopaticle–graphene nanodisk–quantum dot hybrid system

## Abstract

Hybrid nanoplasmonic systems can provide a promising platform of potential nonlinear applications due to the enhancement of optical fields near their surfaces in addition to the control of strong light–matter interactions they can afford. We theoretically investigated the optical multistability of a probe field that circulated along a unidirectional ring cavity containing a metal nanoparticle–graphene nanodisk–quantum dot hybrid system; the quantum dot was modeled as a three-level atomic system of Lambda configuration interacting with probe and control fields in the optical region of the electromagnetic spectrum. We show that the threshold and degree of multistability can be controlled by the geometry of the setup, the size of metal nanoparticles, the carrier mobility in the graphene nanodisk and the detunings of probe and control fields. We found that under electromagnetically-induced transparency conditions the system exhibits enhanced optical multistability with an ultralow threshold in the case of two-photon resonance with high carrier mobility in the graphene nanodisk. Moreover, we calculated the limits of the controllable parameters within which the switching between optical multistability and bistability can occur. We show that our proposed hybrid plasmonic system can be useful for efficient all-optical switches and logic-gate elements for quantum computing and quantum information processing.

## 1. Introduction

Optical bistability at the nanoscale is an attractive research field due to the interesting phenomena it encompasses resulting from controlling light with light [1], and its promising potential applications, including optical memories [2,3,4], optical transistors [5] and all-optical switches [6,7]. Specifically, optical bistability is a nonlinear optical effect arising from third-order nonlinear susceptibility in which the refractive index depends on the light intensity exhibiting self-Kerr nonlinearity [8]. A system is said to be bistable if it has two output states corresponding to the same value of input intensity. This requires an internal feedback mechanism provided by a Kerr nonlinear medium situated inside an optical cavity that enhances the light–matter interaction [7,9].

However, increasing the number of stable output states against a specific input optical state, i.e., optical multistability, can be more attractive than binary optical stability for many applications, such as all-optical switching [10,11,12], quantum computing and quantum information processing [13]. Thus, the optical multistability has been extensively studied in multi-level atomic systems with different configurations inside an optical cavity via the interactions between the nonlinear medium and two optical fields [12,14,15,16].

As a promising controllable platform for the nonlinear applications, hybrid plasmonic systems represent good candidates to demonstrate optical multistability [17,18,19,20,21]. To be of practical interest, the threshold value of input power for multistability should be small [9]. Thanks to the enhanced optical intensities in plasmonic nanocomposites, the desired threshold power required to obtain nonlinear effects is relatively small [20,22]. Moreover, multi-level atomic systems, under coherent excitation resulting in electromagnetically induced transparency (EIT), exhibit an enhanced third-order nonlinear response that could be employed for optical bistability and multistability [20,23,24,25]. For metal nanoparticle (MNP)–quantum dot (QD) hybrid systems, it has been shown that enhanced optical bistability can be controlled by the center-to-center distances between MNP and QD [22,26].

On the other hand, due to the unique nonlinear optical properties resulting from the linear dispersion relation near Dirac points, graphene has remarkably large third-order nonlinear optical susceptibility [27,28]. Recently, Dai X. et al. proposed a modified Kretchmann–Reather configuration to realize low threshold optical bistable devices at terahertz frequencies by using a plasmonic structure with an insertion of graphene [29]. Moreover, it has been experimentally shown that surface plasmons of graphene can be used as an internal feedback to demonstrate an ultralow threshold optical bistability due to the large nonlinear response exhibited by plasmonic structures [30]. It has also been shown that controllable switching between optical bistability and optical multistability is feasible via frequency detunings of probe and control fields in a graphene monolayer system driven by an elliptically polarized control field and a right-hand circularly polarized probe field [31]. Additionally, the optical bistability has been investigated in graphene multilayer systems. It was found that increasing the sheet numbers could lead to large bistability loop width [32]. Recently, T. Naseri et. al. have theoretically investigated THz optical bistability of graphene-coated, cylindrical, core-shell gold nanoparticles. Their hybrid system has exhibited switching between optical bistability and multistability that can be achieved by controlling the Fermi energy and relaxation time of graphene [33].

Interestingly, it has been theoretically shown that metal nanoparticle–graphene nanodisk– quantum dot hybrid systems can demonstrate a controllable giant self-Kerr nonlinearity under EIT conditions with low light intensity [20]. Specifically, it has been found that the magnitude and sign of the nonlinear refractive index can be controlled by the geometry of the hybrid system, Rabi frequency of the control field and detuning of both probe and control fields. Thus, with this novel hybrid plasmonic system, it is expected to obtain low threshold optical bistability and multistability.

**Figure 1:**
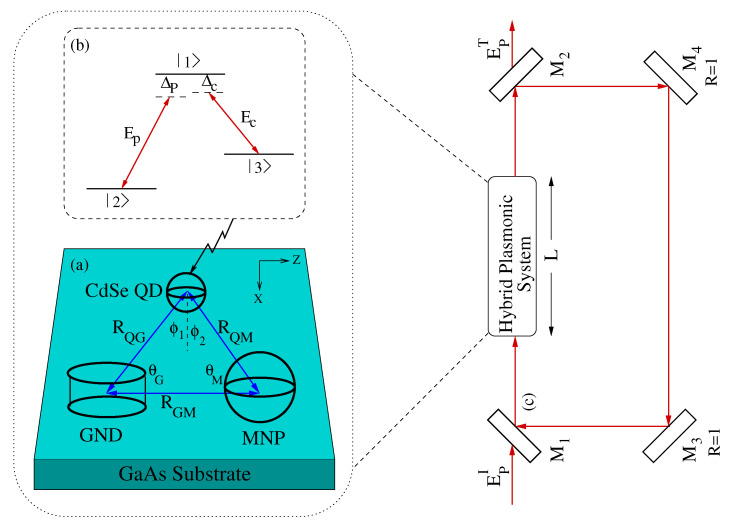
(**a**) The MNP–GND–QD hybrid system setup. (**b**) Λ-type atomic configuration of the QD. (**c**) Unidirectional ring optical cavity having four mirrors and the MNP–GND–QD hybrid system of length *L*. The mirrors $M_{3}$ and $M_{4}$ are perfect mirrors. The incident and transmitted fields are denoted by $E_{p}^{I}$ and $E_{p}^{T}$ respectively.

In this work, we investigated the optical multistability in the metal nanoparticle–graphene nanodisk–quantum dot (MNP–GND–QD) hybrid system depicted in Figure 1, in a unidirectional ring cavity under EIT conditions where the quantum dot is modeled as a three level atomic system of Lambda configuration interacting with probe and control fields under the rotating wave approximation. The ranges of the system parameters were explored to optimize the optical multistability in such a novel system.

## 2. Theoretical Model

We consider the MNP–GND–QD hybrid system deposited on a gallium arsenide (GaAs) substrate as illustrated in Figure 1 The QD is modeled as a three level atomic system of Λ configuration, where the transition |1〉↔|2〉 of dipole moment $\mu_{12}$ is induced by the probe field of frequency $\omega_{p}$, Rabi frequency $\Omega_{p}$ and detuning of $\Delta_{p}=\omega_{12}-\omega_{p}$, whereas the control field of frequency $\omega_{c}$, Rabi frequency $\Omega_{c}$ and detuning of $\Delta_{c}=\omega_{13}-\omega_{c}$ is driving the transition |1〉↔|3〉 of dipole moment $\mu_{13}$. Note that the dipole moment $\mu_{12}$ ($\mu_{13}$) lies along the x (z) direction, so that the probe (control) field is applied along the x (z) direction. By analyzing the dipole–dipole interaction between the components of the system within the near field approximation, and solving the Lindblad quantum master equation using a Hamiltonian given in terms of the dipole field felt by the QD with two-photon detuning, $\Delta_{2}=\Delta_{p}-\Delta_{c}$, in the rotating wave approximation, one can get the following equations of motion for the density matrix elements [34]:

(1a) $$\begin{array}{cc} {\dot{\rho }}_{13}= & -\left[\left(\frac{\gamma_{13}}{2}+\frac{\gamma_{12}}{2}\right)+i\left(\Delta_{c}-\Lambda_{z}\left(\rho_{33}-\rho_{11}\right)\right)\right]\rho_{13}\\ & +i\Omega_{c}\left(\Pi_{z}+\Phi_{z}\right)\left(\rho_{33}-\rho_{11}\right)+i\left[\Omega_{p}\left(\Pi_{x}+\Phi_{x}\right)+\Lambda_{x}\rho_{12}\right]\rho_{23}, \end{array}$$

(1b) $$\begin{array}{cc} {\dot{\rho }}_{12}= & -\left[\left(\frac{\gamma_{13}}{2}+\frac{\gamma_{12}}{2}\right)+i\left(\Delta_{p}-\Lambda_{x}\left(\rho_{22}-\rho_{11}\right)\right)\right]\rho_{12}\\ & +i\Omega_{p}\left(\Pi_{x}+\Phi_{x}\right)\left(\rho_{22}-\rho_{11}\right)+i\left[\Omega_{c}\left(\Pi_{z}+\Phi_{z}\right)+\Lambda_{z}\rho_{13}\right]\rho_{32}, \end{array}$$

(1c) $$\begin{array}{cc} {\dot{\rho }}_{32}= & -\left(\frac{\gamma_{32}}{2}+i\Delta_{2}\right)\rho_{32}+i\left[\Omega_{c}^{*}\left(\Pi_{z}^{*}+\Phi_{z}^{*}\right)+\Lambda_{z}^{*}\rho_{31}\right]\rho_{12}\\ & -i\left[\Omega_{p}\left(\Pi_{x}+\Phi_{x}\right)+\Lambda_{x}\rho_{12}\right]\rho_{31}, \end{array}$$

(1d) $$\begin{array}{cc} {\dot{\rho }}_{11}= & -\left(\gamma_{12}+\gamma_{13}\right)\rho_{11}+i\left[\Omega_{c}\left(\Pi_{z}+\Phi_{z}\right)+\Lambda_{z}\rho_{13}\right]\rho_{31}\\ & +i\left[\Omega_{p}\left(\Pi_{x}+\Phi_{x}\right)+\Lambda_{x}\rho_{12}\right]\rho_{21}+c.c., \end{array}$$

(1e) $${\dot{\rho }}_{22}=\gamma_{12}\rho_{11}+\gamma_{32}\left(\rho_{33}-\rho_{22}\right)-i\left[\Omega_{p}\left(\Pi_{x}+\Phi_{x}\right)+\Lambda_{x}\rho_{12}\right]\rho_{21}+c.c.,$$

(1f) $${\dot{\rho }}_{33}=\gamma_{13}\rho_{11}+\gamma_{32}\left(\rho_{22}-\rho_{33}\right)-i\left[\Omega_{c}\left(\Pi_{z}+\Phi_{z}\right)+\Lambda_{z}\rho_{13}\right]\rho_{31}+c.c.,$$

In Equations (1), $\gamma_{1i}$ represents the spontaneous decay rate of the QD while $\gamma_{32}$ stands for the lower states’ dephasing. It is remarkable that the Rabi frequency of probe field (control field) is enhanced by a factor $|\Pi_{x}+\Phi_{x}|$ ($|\Pi_{z}+\Phi_{z}|$), whereas $\Lambda_{x}$ ($\Lambda_{z}$) enhances the dephasing rate induced by the probe (control) field. The enhancement factors Π, Φ and Λ resulting from the dipole–dipole interaction are given for the system shown in Figure 1 by [34]:

(2a) $$\begin{array}{c} \Pi_{x}=\frac{1}{4\pi \epsilon^{*}}\left[\frac{\alpha_{G}^{x}\left(3cos\phi_{1}-1\right)}{R_{QG}^{3}}+\frac{\alpha_{M}\left(3cos\phi_{2}-1\right)}{R_{QM}^{3}}\right], \end{array}$$

(2b) $$\Phi_{x}=\frac{-\alpha_{G}^{x}\alpha_{M}}{{\left(4\pi \epsilon^{*}\right)}^{2}R_{GM}^{3}}\left[\frac{3cos\phi_{1}-1}{R_{QG}^{3}}+\frac{3cos\phi_{2}-1}{R_{QM}^{3}}\right],$$

(2c) $$\Lambda_{x}=\frac{\mu_{12}^{2}}{{\left(4\pi \epsilon^{*}\right)}^{2}\hbar \epsilon_{0}\epsilon_{b}}\left[\frac{\alpha_{G}^{x}{\left(3cos\phi_{1}-1\right)}^{2}}{R_{QG}^{6}}+\frac{\alpha_{M}{\left(3cos\phi_{2}-1\right)}^{2}}{R_{QM}^{6}}\right],$$

(2d) $$\Pi_{z}=\frac{1}{4\pi \epsilon^{*}}\left[\frac{\alpha_{G}^{z}\left(3cos\theta_{G}-1\right)}{R_{QG}^{3}}+\frac{\alpha_{M}\left(3cos\theta_{M}-1\right)}{R_{QM}^{3}}\right],$$

(2e) $$\Phi_{z}=\frac{2\alpha_{G}^{z}\alpha_{M}}{{\left(4\pi \epsilon^{*}\right)}^{2}R_{GM}^{3}}\left[\frac{3cos\theta_{G}-1}{R_{QG}^{3}}+\frac{3cos\theta_{M}-1}{R_{QM}^{3}}\right],$$

(2f) $$\Lambda_{z}=\frac{\mu_{13}^{2}}{{\left(4\pi \epsilon^{*}\right)}^{2}\hbar \epsilon_{0}\epsilon_{b}}\left[\frac{\alpha_{G}^{z}{\left(3cos\theta_{G}-1\right)}^{2}}{R_{QG}^{6}}+\frac{\alpha_{M}{\left(3cos\theta_{M}-1\right)}^{2}}{R_{QM}^{6}}\right],$$

$\alpha_{G}^{x}$ ($\alpha_{G}^{z}$) is the shape-dependent polarizability of GND induced by x (z) polarized field while $\alpha_{M}$ represents the polarizability of MNP given in terms of its volume and dielectric constant of the metal $\epsilon_{M}$ and the background $\epsilon_{b}$ [35]. The center-to-center distances $R_{QG}$, $R_{QM}$ and $R_{GM}$ are governed by the triangle law:

(3a) $$\begin{array}{c} R_{QG}=\left(\frac{\sin\theta_{M}}{\sin\theta_{Q}}\right)R_{GM} \end{array}$$

(3b) $$R_{QM}=\left(\frac{\sin\theta_{G}}{\sin\theta_{Q}}\right)R_{GM}$$

The MNP–GND–QD hybrid system sample of length *L* is placed in a unidirectional ring cavity, having four mirrors as shown in Figure 1. Mirrors $M_{1}$ and $M_{2}$ have identical reflection *R* and transmission *T* coefficients, where R+T=1. On the other hand, mirrors $M_{3}$ and $M_{4}$ are considered to be perfect reflectors to simplify optical multistability analysis. The MNP–GND–QD hybrid sample is situated in one of the arms of the cavity whose dynamics is described by the time evolution of the density matrix elements given by Equation (1). By using this standard model [36], the probe field passes through the nonlinear medium of length *L* from the partially transparent mirror $M_{1}$ and is redirected back to the entry point by the system of mirrors illustrated in Figure 1. Therefore, the optical stability can be analyzed by measuring the input and output beams generated by the two partially transmitting mirrors, $M_{1}$ and $M_{2}$. Note that only the probe field acts as a cavity field and circulates inside the cavity, whereas the control field does not circulate inside the cavity. Therefore, the induced atomic polarization responsible for the optical multistability is $P\left(\omega_{p}\right)=N\mu_{21}\rho_{21}$, where *N* is the atomic number density, $\mu_{12}$ is the transition dipole matrix element for the probe field transition that induces atomic coherence $\rho_{21}$. The propagation of the probe field $E_{p}$ in the unidirectional optical ring cavity is governed by the following Maxwell equation under the slowly varying envelope approximation where $d^{2}/dz^{2}$ can be neglected [8]:

(4) $$\frac{\partial E_{p}}{\partial t}+c\frac{\partial E_{p}}{\partial z}=i\frac{\omega_{p}P\left(\omega_{p}\right)}{2\epsilon_{0}}$$

By inserting the relation of polarization induced by the probe field into Equation (4) we obtain at steady state:

(5) $$\frac{\partial E_{p}}{\partial z}=i\frac{N\omega_{p}\mu_{21}\rho_{21}}{2c\epsilon_{0}}$$

For a perfectly tuned ring cavity, the incident ($E_{p}^{I}$) and the transmitted ($E_{p}^{T}$) probe fields obey the following boundary conditions in the steady state limit [1]:

(6a) $$\begin{array}{c} E_{p}\left(L\right)=\frac{E_{p}^{T}}{\sqrt{T}} \end{array}$$

(6b) $$E_{p}\left(0\right)=\sqrt{T}E_{p}^{I}+RE_{p}^{T}\left(L\right)$$

Solving the differential equation Equation (5) using the boundary conditions given by Equation (6) leads to:

(7) $$Y=X-iC\rho_{21}$$

where $Y=\mu_{12}E_{p}^{I}/\hbar \gamma_{12}\sqrt{T}$ and $X=\mu_{12}E_{p}^{T}/\hbar \gamma_{12}\sqrt{T}$ are normalized incident and output fields respectively given in a dimensionless form. Note that the second term of Equation (7) describes the feedback mechanism provided by the system of mirrors that is essential to obtain optical bistability and multistability, where *C* is the cooperation parameter that is proportional to the density of absorbing atoms in the cavity. More precisely, C=αL/2T, where $\alpha =N\omega_{p}\mu_{12}^{2}/c\epsilon_{0}\hbar \gamma_{12}$ is the absorption coefficient.

## 3. Results and Discussion

In order to analyze the optical multistability in the MNP–GND–QD hybrid system and optimize its threshold in this system, we used the same parameters as in reference [20], wherein it was shown that the MNP–GND–QD hybrid system can demonstrate giant self-Kerr nonlinearity under EIT conditions. Due to the unique properties of GND plasmons, including the high mobility and relatively long propagation distances [37], we adjusted the energy of its plasmons to be resonant with the exciton of the QD. Consider GND of radius 7 nm and thickness of 0.5 nm at Fermi energy 1.36 eV and temperature 300 K, and carrier mobility $10^{4}$ cm^2^/Vs. With these parameters of GND deposited on GaAs substrate, we get plasmon resonances along x and z directions, i.e., $\hbar \omega_{sp}^{x}=2.17$ eV and $\hbar \omega_{sp}^{z}=0.6418$ eV. To support the plasmons of GND and provide more options to control the system, we used a spherical silver nanoparticle of $\epsilon_{\infty }=5.7$ [38], and plasma frequency of $\omega_{pl}=1.36\times 10^{16}$ s^−1^, and damping rate of plasmons of $\gamma_{M}=10^{14}$ s^−1^. CdSe self-assembled QD of $N=10^{20}$ m^−3^ was chosen to compensate for the losses of plasmons due to its optical emission band, which was near resonant with $\hbar \omega_{sp}^{x}=2.17$ eV in GND induced by x-polarized probe field. The physical parameters used in the numerical simulation are summarized in Table 1.

In the following we investigate the controlling of optical multistability by the parameters of the system, including the inclination angle of the MNP; the edge-to-edge distances between GND and MNP; and the size of MNP. The effects of the detunings of the probe and control fields were examined in addition to the carrier mobility in GND. We compared the results under EIT conditions, i.e., $\Omega_{c}\geq \gamma_{12}$, and $\gamma_{23}\ll \gamma_{12}$, to when these conditions were not fulfilled in order to optimize the threshold of optical multistability in the proposed MNP–GND–QD hybrid system.

**Figure 2:**
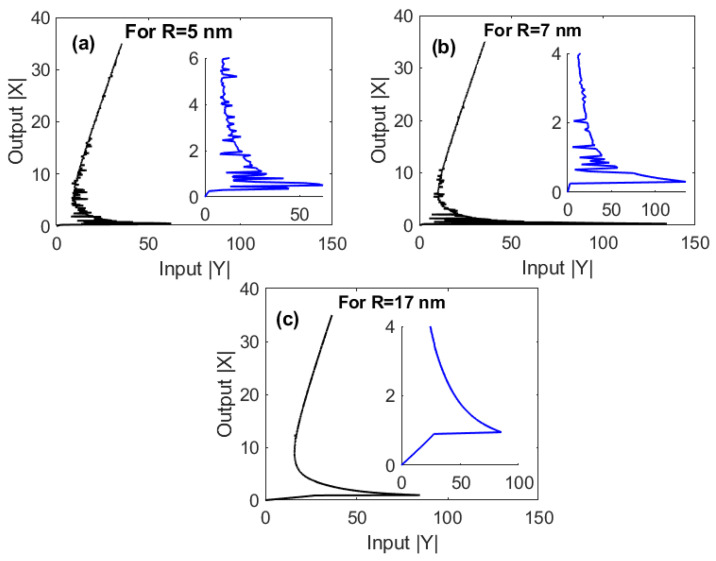
The output versus the input for different values of the edge-to-edge distances (R) between GND and MNP under electromagnetically induced transparency (EIT) conditions; $\frac{\Omega_{c}}{\gamma_{12}}=2$, and $\frac{\gamma_{32}}{\gamma_{12}}=0.4$. The other parameters were $\frac{\Delta_{p}}{\gamma_{12}}=1$, $\frac{\Delta_{c}}{\gamma_{12}}=0$, $R_{M}=15$ nm, $\theta_{M}=0.4$ rad, $\theta_{G}=1$ rad and C=121.36, and the mobility of GND was $\mu =10^{4}$ cm^2^/Vs.

**Figure 3:**
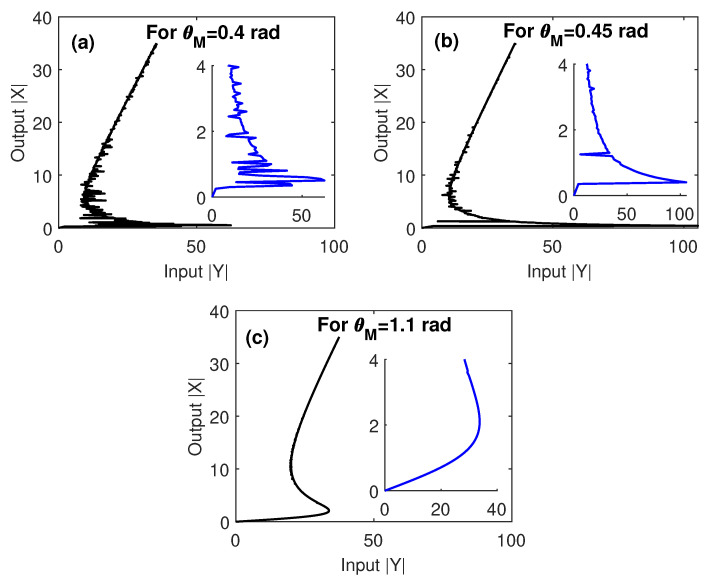
The output versus the input for different values of the inclination angle of MNP with respect to QD, under EIT conditions; $\frac{\Omega_{c}}{\gamma_{12}}=2$, and $\frac{\gamma_{32}}{\gamma_{12}}=0.4$. The other parameters were $\frac{\Delta_{p}}{\gamma_{12}}=1$, $\frac{\Delta_{c}}{\gamma_{12}}=0$, $R_{M}=15$ nm, R=5 nm, $\theta_{G}=1$ rad and C=121.36, and the mobility of GND was $\mu =10^{4}$ cm^2^/Vs.

**Figure 4:**
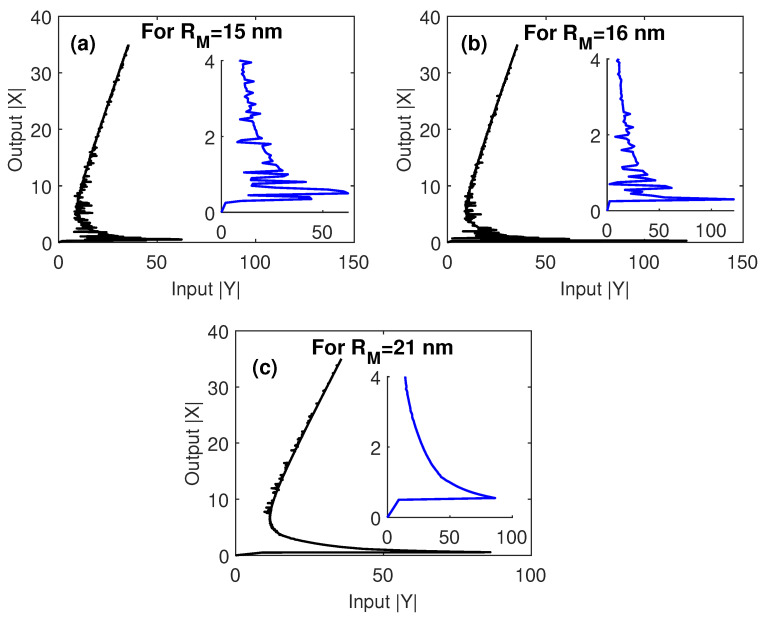
The output versus the input for different values of the MNP size, under EIT conditions; $\frac{\Omega_{c}}{\gamma_{12}}=2$, and $\frac{\gamma_{32}}{\gamma_{12}}=0.4$. The other parameters were $\frac{\Delta_{p}}{\gamma_{12}}=1$, $\frac{\Delta_{c}}{\gamma_{12}}=0$, R=5 nm, $\theta_{M}=0.4$ rad, $\theta_{G}=1$ rad and C=121.36, and the mobility of GND was $\mu =10^{4}$ cm^2^/Vs.

**Figure 5:**
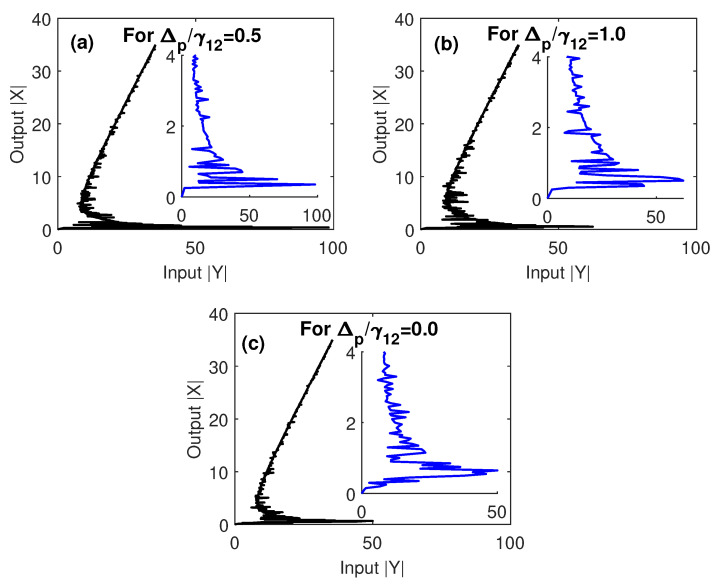
The output versus the input for different values of $\frac{\Delta_{p}}{\gamma_{12}}$, under EIT conditions; $\frac{\Omega_{c}}{\gamma_{12}}=2$, and $\frac{\gamma_{32}}{\gamma_{12}}=0.4$. The other parameters were $\frac{\Delta_{c}}{\gamma_{12}}=0$, R=5 nm, $\theta_{M}=0.4$ rad, $\theta_{G}=1$ rad and C=121.36, and the mobility of GND was $\mu =10^{4}$ cm^2^/Vs.

**Figure 6:**
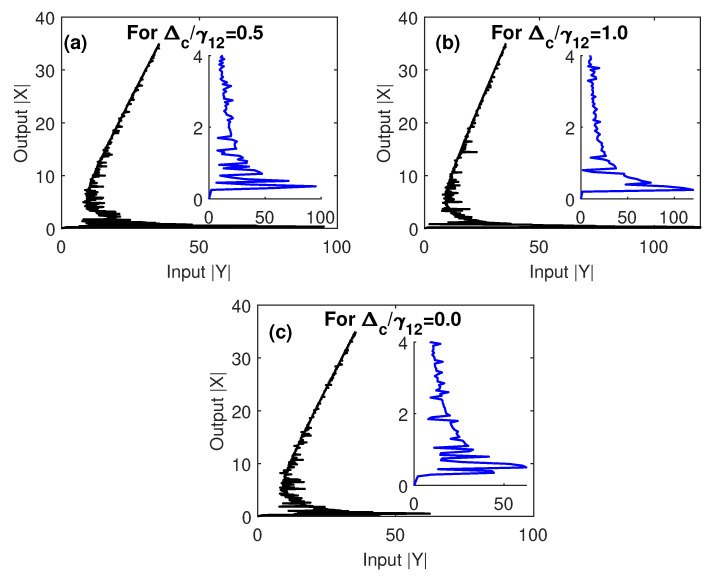
The output versus the input for different values of $\frac{\Delta_{c}}{\gamma_{12}}$, under EIT conditions; $\frac{\Omega_{c}}{\gamma_{12}}=2$, and $\frac{\gamma_{32}}{\gamma_{12}}=0.4$. The other parameters were $\frac{\Delta_{p}}{\gamma_{12}}=1$, R=5 nm, $\theta_{M}=0.4$ rad, $\theta_{G}=1$ rad and C=121.36, and the mobility of GND was $\mu =10^{4}$ cm^2^/Vs.

**Figure 7:**
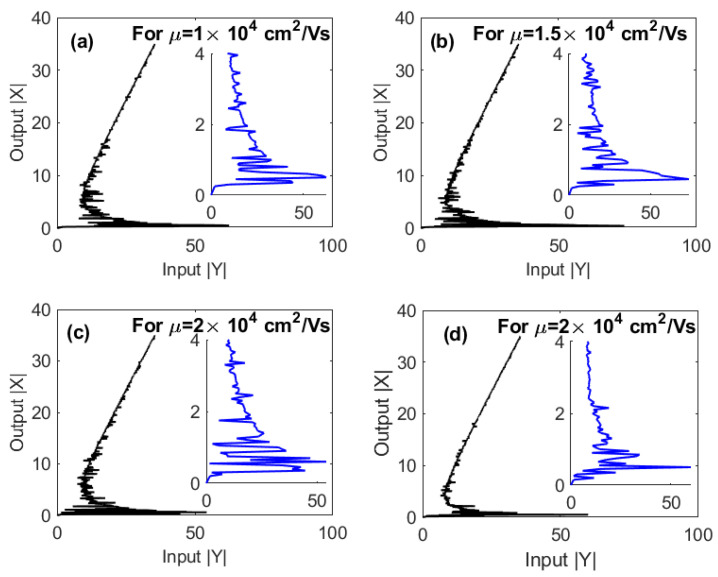
The output versus the input for different values of the mobility of GND under EIT conditions; $\frac{\Omega_{c}}{\gamma_{12}}=2$, and $\frac{\gamma_{32}}{\gamma_{12}}=0.4$. The other parameters were $\frac{\Delta_{p}}{\gamma_{12}}=1$; (**a**–**c**) $\frac{\Delta_{p}}{\gamma_{12}}=0$; (**d**) $\frac{\Delta_{c}}{\gamma_{12}}=0$, R=5 nm, $\theta_{M}=0.4$ rad, $\theta_{G}=1$ rad and C=121.36.

Under EIT conditions, i.e., $\frac{\Omega_{c}}{\gamma_{12}}=2$ and $\frac{\gamma_{32}}{\gamma_{12}}=0.4$, we firstly examine the effects of the edge-to-edge distances between GND and MNP (R) on the output–input relationship, as illustrated in Figure 2. It is remarkable that our proposed hybrid plasmonic system supports the optical multistability due to giant self-Kerr nonlinearity demonstrated by our proposed system, as shown in reference [20]. Obviously, the degree of multistability decreases as (R) increases. With increasing (R), larger input field is needed to achieve the optical multistability. Specifically, increasing *R* from 5 nm (Figure 2a) to 7 nm (Figure 2b) leads to increasing the threshold of optical multistability from 20 W cm^−2^ to 188 W cm^−2^. Interestingly, these values of the threshold are ultralow compared to those that have recently been obtained for some graphene plasmonic systems [29,39,40]. In fact, increasing the edge-to-edge distances between MNP and GND will lead to increased distances between GND and QD, as noted from Equation (3), which negatively affects the energy transfer between their optical excitations. Interestingly, a switching between optical multistability and bistability can be induced at relatively large edge-to-edge distances between GND and MNP that represent 0.6296 of the center-to-center distance between MNP and GND $\left(R_{M}+R+L_{z}\right)$, as illustrated in Figure 2c.

In order to compare the sensitivity of the optical multistability to the geometry of the system via manipulating the edge-to-edge distances between MNP and GND, and the inclination angle of the two components with respect to QD, we checked in Figure 3 the output–input relationship at different values of $\theta_{M}$. We observed that a small $\theta_{M}$ led to a large number of loops of multistability with relativity low threshold. As $\theta_{M}$ increases, the degree of multistability decreases, while the threshold increases. It is clear that the optical multistability of the MNP–GND–QD hybrid system exhibits high sensitivity to $\theta_{M}$, since increasing the latter by 0.05 rad leads to doubling the optical multistability threshold. Moreover, a transition between optical multistability and bistability is shown at relativity large $\theta_{M}$, where $\theta_{M}>\theta_{G}$. This is due to the large center-to-center distances between GND and QD obtained in this case as noted by Equation (3).

The effect of the size of MNP is investigated in Figure 4. The threshold size of MNP required to get a high degree of multistability with a low threshold is that of radius equal to 0.555 of the center-to-center distance between MNP and GND; i.e., $R_{M}=15$ nm, which has been used in Figure 4. Increasing the size of MNP leads to decreasing the degree of optical multistability and increasing its threshold. This can be attributed to the large associated center-to-center distances between MNP and GND that strongly decrease the enhancement factor (Λ), as observed by Equation (2), in addition to the large corresponding center-to-center distances between GND and QD that lead to a reduction of the energy transfer between the two components. For this reason, when we set the size of MNP to 0.6363 of the center-to-center distances between MNP and GND, we observed a switching between optical multistability and bistability, as shown in Figure 4c.

It is worth noting here that the tolerance intervals for the critical parameters that our system can afford without losing the optical multistability performance are almost reasonable. In particular, the system still demonstrates optical multistability with edge-to-edge distances between MNP and GND (R) in the range 5–17 nm (Figure 2), an inclination angle of MNP ($\theta_{M}$) in the range 0.4–1.1 rad (Figure 3) and a radius of MNP $R_{M}$ in the range 15–21 nm (Figure 4).

To figure out how the optical multistability of our proposed system can be controlled by the detunings of probe and control fields, we plot in Figure 5 and Figure 6 the relationships between the output and input fields at different values of $\Delta_{p}$ and $\Delta_{c}$. Figure 5 shows the control of optical multistability of the MNP–GND–QD hybrid system by the detuning of the probe field. It is clear from Figure 5c that, when the probe field is resonant with the atomic transition |1〉↔|2〉, the threshold of multistability is significantly reduced while the number of its loops is increased. This seems reasonable since a strong coupling between GND and QD is induced at resonance resulting in the enhancement of the nonlinearity of the proposed hybrid system and a reduction in the input field required to trigger the optical multistability. Moreover, if the damping rate of excited state (|1〉) exceeds the detuning of the probe field, the threshold of optical multistability is relatively large (Figure 5a) and decreases as the ratio $\Delta_{p}/\gamma_{12}$ increases (Figure 5b).

On the other hand, we examine in Figure 6 the extent to which the optical multistability of the proposed MNP–GND–QD can be controlled by the detuning of the control field that does not circulate in the optical ring cavity. Compared to Figure 5, with a resonant control field, similar results were found, but different behavior was observed for off-resonant control field. Specifically, we observed that the threshold of optical multistability was relatively small for the case of $\Delta_{c}<\gamma_{12}$ (Figure 6a). Interestingly, the case of resonant probe and control fields that is depicted in Figure 5c shows an extremely low threshold of optical multistability due to the enhanced energy transfer associated with two-photon detuning [41].

One of the unique properties of graphene that controls the magnitude of the extinction cross-section and the energy of graphene plasmons is the mobility of graphene charge carriers (μ). However, due to the high carrier mobility in graphene, the different values of (μ) with the same order of magnitude can lead to the same energy of plasmons. Therefore, we can safely change the values of the mobility while ensuring that the energy of GND plasmons remains resonant with excitons in the QD. Figure 7 shows the effect of the graphene carrier mobility on the optical multistability. It can be seen that the number of loops increases while the threshold of multistability decreases as the mobility of graphene increases. This result can be understood based on the relation between the relaxation rate of graphene plasmons and the mobility of its charge carriers, i.e., $\gamma_{G}=ev_{F}^{2}/\mu E_{F}$. In other words, higher mobility means lower damping rate of graphene plasmons that can enhance the nonlinearity of the system.

Based on the above results for optical multistability under EIT conditions, it is remarkable that an extremely low threshold of optical multistability can be obtained for a resonant probe field that induces the plasmons of a relatively large-mobility GND as shown in Figure 5c and Figure 7d, compared to those have been found for MNP-QD hybrid system [26].

**Figure 8:**
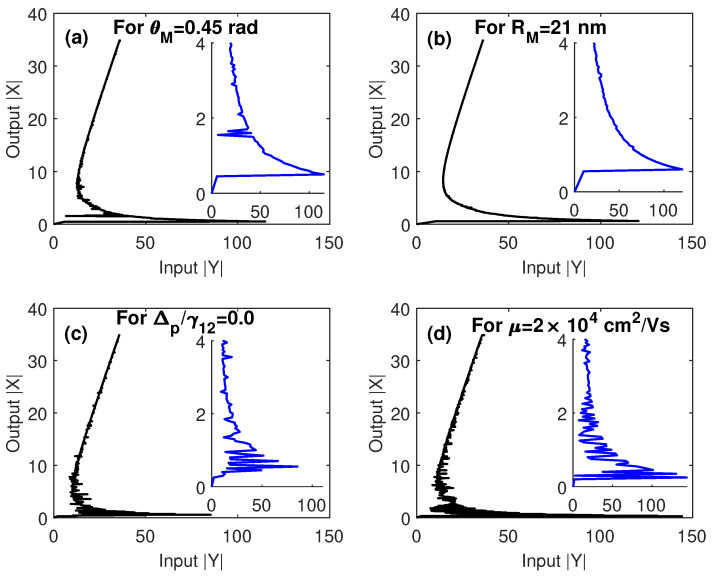
The output versus the input out of EIT conditions; $\frac{\Omega_{c}}{\gamma_{12}}=0.5$, and $\frac{\gamma_{32}}{\gamma_{12}}=1$. The other parameters were $\frac{\Delta_{p}}{\gamma_{12}}=1$ in (**a**,**b**,**d**), $\frac{\Delta_{c}}{\gamma_{12}}=0$, R=5 nm, $\theta_{M}=0.4$ rad, $\theta_{G}=1$ rad and C=121.36, and the mobility of GND was $\mu =10^{4}$ cm^2^/Vs in (**a**–**c**).

Finally, in Figure 8 we show some of the above multistability curves when the conditions of EIT are not fulfilled; i.e., $\frac{\Omega_{c}}{\gamma_{12}}=0.5$, and $\frac{\gamma_{32}}{\gamma_{12}}=1$. From these results, it can be seen that under conditions of EIT, the multistability threshold is significantly reduced because of the enhanced nonlinearity induced by steep dispersion associated with EIT. On the other hand, the limits of the parameters within which the switching between optical multistability and bistability can occur, are unaffected, as shown in Figure 8b. Taking into account all cases examined in Figure 8, we can conclude that when EIT conditions are not fulfilled, we can obtain a relatively low threshold for multistability only for a resonant probe field (Figure 8c). This is apparently due to the strong energy transfer between plasmons in GND and excitons in the QD for resonant probe field.

## 4. Conclusions

We studied the optical multistability induced in a unidirectional ring cavity due to the interactions in a MNP–GND–QD hybrid system with probe and control fields in the optical range of the electromagnetic spectrum, where the QD is considered as a three-level atomic system of Lambda configuration. We have found that our proposed system can support controllable optical multistability resulting from the giant self-Kerr nonlinearity demonstrated by this system. Moreover, the extremely low threshold and high degree of optical multitability were obtained at two-photon resonance with high mobility GND under EIT conditions. We calculated the limits of the geometrical structure parameters within which the switching between optical multistability and optical bistability can occur. Interestingly, this optical switching can also be controlled by the detuning and Rabi frequency of the probe and control fields. Therefore, the values related to the geometrical structure and materials parameters of the hybrid system as well as the power and detuning of the probe and control fields, turned out to be well within the limits of current materials technology.

Our results demonstrate that MNP–GND–QD hybrid systems are unique platforms on which to observe controllable optical multistability that can be switched to optical bistability. Thus, the results of our work may contribute to a deeper insight on the control of light by light in such novel systems that can be used to build efficient optical multistable nanoswitches and logic-gate elements for quantum memories. To achieve a more comprehensive understanding of this unique system and its potential applications, future works could include investigations into the optical multistability in MNP–GND–QD hybrid systems with typical values of Fermi energy that are suitable for operation in infrared light.

## Figures and Tables

**Table 1:** The physical parameters used in the numerical simulations.

System Component (s)	Parameter (Symbol)	Value
The Metal Nanoparticle (MNP)	High-frequency dielectric constant ($\epsilon_{\infty }$)	$\epsilon_{\infty }=5.7$
	Plasma frequency ($\omega_{{}_{pl}}$)	$\omega_{{}_{pl}}=1.36\times 10^{16}$ (s^−1^)
	Damping rate of plasmon(s) ($\gamma_{{}_{M}}$)	$\gamma_{{}_{M}}=10^{14}$ (s^−1^)
The Graphene Nanodisk (GND)	Thickness ($L_{x}$)	$L_{x}=0.5$ (nm)
	Radius ($L_{z}$)	$L_{z}=7$ (nm)
	Mobility (μ)	$\mu =10^{4}$ (cm^2^/Vs)
	Fermi energy ($F_{E}$)	$F_{E}=1.36$ (eV)
The CdSe Quantum Dot (CdSe QD)	Dielectric constant ($\epsilon_{{}_{q}}$)	$\epsilon_{{}_{q}}=6.5$
	Dipole moment transition ($\mu_{ij}$)	$\mu_{12}=\mu_{13}=0.1$ (e nm)
	Atomic number density (*N*)	$N=10^{20}$ (m^−3^)
The GaAs substrate (Background)	Dielectric constant ($\epsilon_{{}_{b}}$)	$\epsilon_{{}_{b}}=12.9$

## Abbreviations

The following abbreviations are used in this manuscript:

MNP	Metal nanoparticle
GND	Graphene nanodisk
QD	Quantum dot
EIT	Electromagnetically induced transparency

## References

[1] Gibbs H. (1985). Optical Bistability: Controlling Light with Light.

[2] Abraham E., Smith S. (1982). Optical bistability and related devices. Rep. Prog. Phys..

[3] Li Y.N., Chen Y.Y., Wan R.G., Yan H.W. (2019). Dynamical switching and memory via incoherent pump assisted optical bistability. Phys. Lett. A.

[4] Smith S. (1986). Optical bistability, photonic logic, and optical computation. Appl. Opt..

[5] Assanto G., Wang Z., Hagan D., VanStryland E. (1995). All-optical modulation via nonlinear cascading in type II second-harmonic generation. Appl. Phys. Lett..

[6] Mazurenko D.A., Kerst R., Dijkhuis J., Akimov A., Golubev V., Kurdyukov D., Pevtsov A., Sel’Kin A. (2003). Ultrafast optical switching in three-dimensional photonic crystals. Phys. Rev. Lett..

[7] Singh M.R. (2020). Theory of all-optical switching based on the Kerr nonlinearity in metallic nanohybrids. Phys. Rev. A.

[8] Boyd R.W. (2003). Nonlinear Optics.

[9] Joshi A., Xiao M. (2006). Controlling nonlinear optical processes in multi-level atomic systems. Prog. Opt..

[10] Nobrega K., Da Silva M., Sombra A. (2000). Multistable all-optical switching behavior of the asymmetric nonlinear directional coupler. Opt. Commun..

[11] Miroshnichenko A.E., Brasselet E., Kivshar Y.S. (2008). All-optical switching and multistability in photonic structures with liquid crystal defects. Appl. Phys. Lett..

[12] Sheng J., Khadka U., Xiao M. (2012). Realization of all-optical multistate switching in an atomic coherent medium. Phys. Rev. Lett..

[13] Martellucci S., Chester A.N. (2012). Nonlinear Optics and Optical Computing.

[14] Wang Z., Xu M. (2009). Control of the switch between optical multistability and bistability in three-level V-type atoms. Opt. Commun..

[15] Sahrai M., Hamedi H., Memarzadeh M. (2012). Kerr nonlinearity and optical multi-stability in a four-level Y-type atomic system. J. Mod. Opt..

[16] Asadpour S.H., Soleimani H.R. (2016). Optical bistability and multistability in a four-level quantum system in the presence of plasmonic nanostructure. Phys. E Low-Dimens. Syst. Nanostructures.

[17] Chen H., Ren J., Gu Y., Zhao D., Zhang J., Gong Q. (2015). Nanoscale Kerr nonlinearity enhancement using spontaneously generated coherence in plasmonic nanocavity. Sci. Rep..

[18] Terzis A., Kosionis S., Boviatsis J., Paspalakis E. (2016). Nonlinear optical susceptibilities of semiconductor quantum dot–metal nanoparticle hybrids. J. Mod. Opt..

[19] Ren J., Chen H., Gu Y., Zhao D., Zhou H., Zhang J., Gong Q. (2016). Plasmon-enhanced Kerr nonlinearity via subwavelength-confined anisotropic Purcell factors. Nanotechnology.

[20] Tohari M., Lyras A., AlSalhi M. (2018). Giant Self-Kerr Nonlinearity in the Metal Nanoparticles-Graphene Nanodisks-Quantum Dots Hybrid Systems Under Low-Intensity Light Irradiance. Nanomaterials.

[21] Kosionis S.G., Paspalakis E. (2019). Control of Self-Kerr Nonlinearity in a Driven Coupled Semiconductor Quantum Dot–Metal Nanoparticle Structure. J. Phys. Chem. C.

[22] Bao C., Qi Y., Niu Y., Gong S. (2016). Surface plasmon-assisted optical bistability in the quantum dot-metal nanoparticle hybrid system. J. Mod. Opt..

[23] Wang H., Goorskey D., Xiao M. (2001). Enhanced Kerr nonlinearity via atomic coherence in a three-level atomic system. Phys. Rev. Lett..

[24] Van Doai L., Khoa D.X., Bang N.H. (2015). EIT enhanced self-Kerr nonlinearity in the three-level lambda system under Doppler broadening. Phys. Scr..

[25] Yan X.A., Wang L.Q., Yin B.Y., Song J.P. (2011). Electromagnetically induced transparency and enhanced self-Kerr nonlinearity in a four-level scheme. Optik.

[26] Solookinejad G., Jabbari M., Nafar M., Ahmadi E., Asadpour S. (2018). Incoherent control of optical bistability and multistability in a hybrid system: Metallic nanoparticle-quantum dot nanostructure. J. Appl. Phys..

[27] Hendry E., Hale P.J., Moger J., Savchenko A., Mikhailov S.A. (2010). Coherent nonlinear optical response of graphene. Phys. Rev. Lett..

[28] Cheng J.L., Vermeulen N., Sipe J. (2014). Third order optical nonlinearity of graphene. New J. Phys..

[29] Dai X., Jiang L., Xiang Y. (2015). Low threshold optical bistability at terahertz frequencies with graphene surface plasmons. Sci. Rep..

[30] Sharif M.A., Khodavirdizadeh M., Salmani S., Mohajer S., Ara M.M. (2019). Difference Frequency Generation-based ultralow threshold Optical Bistability in graphene at visible frequencies, an experimental realization. J. Mol. Liq..

[31] Zhang D., Sun Z., Ding C., Yu R., Yang X. (2016). Controllable optical bistability and multistability in a graphene monolayer system. J. Lumin..

[32] Sadeghi M., Ahmadi V. (2018). Multilayer graphene based optical bistability. JOSA B.

[33] Naseri T., Daneshfar N., Moradi-Dangi M., Eynipour-Malaee F. (2018). Terahertz optical bistability of graphene-coated cylindrical core–shell nanoparticles. J. Theor. Appl. Phys..

[34] Tohari M., Lyras A., Alsalhi M. (2019). Ultrafast Energy Transfer in the Metal Nanoparticles-Graphene Nanodisks-Quantum Dots Hybrid Systems. Plasmonics.

[35] Singh J., Williams R.T. (2015). Excitonic and Photonic Processes in Materials.

[36] Bonifacio R., Lugiato L. (1978). Optical bistability and cooperative effects in resonance fluorescence. Phys. Rev. A.

[37] Falkovsky L. (2008). Optical properties of graphene. Journal of Physics: Conference Series.

[38] Johnson P.B., Christy R.W. (1972). Optical constants of the noble metals. Phys. Rev. B.

[39] Guo J., Jiang L., Jia Y., Dai X., Xiang Y., Fan D. (2017). Low threshold optical bistability in one-dimensional gratings based on graphene plasmonics. Opt. Express.

[40] Kar A., Goswami N., Saha A. (2017). Long-range surface plasmon-induced tunable ultralow threshold optical bistability using graphene sheets at terahertz frequency. Appl. Opt..

[41] Berman P.R., Lin C.C., Arimondo E. (2006). Advances in Atomic, Molecular, and Optical Physics.

